# Regular Dental Visits: Influence on Health-Related Quality of Life in 1,607 Patients with Oral Squamous Cell Carcinoma

**DOI:** 10.1155/2017/9638345

**Published:** 2017-05-30

**Authors:** Simon Spalthoff, Henrik Holtmann, Gertrud Krüskemper, Rüdiger Zimmerer, Jörg Handschel, Nils-Claudius Gellrich, Philipp Jehn

**Affiliations:** ^1^Department of Cranio and Maxillofacial Surgery, Hannover Medical School, Carl-Neuberg-Str. 1, 30625 Hannover, Germany; ^2^Department of Cranio and Maxillofacial Surgery, Heinrich Heine University of Düsseldorf, Moorenstr. 5, 40225 Düsseldorf, Germany; ^3^Department of Medical Psychology, Ruhr University of Bochum, Universitätsstr. 150, Building MA 0/145, 44780 Bochum, Germany

## Abstract

**Background:**

The incidence of oral squamous cell carcinoma (OSCC) is in the top 10 of all cancer entities. Regular oral examinations by dentists play an important role in oral cancer prevention.

**Methods:**

Patients with OSCC (*n* = 1,607) and physicians (*n* = 1,489) completed questionnaires during the DÖSAK Rehab Study. The psychosocial and functional factors collected in these questionnaires were assessed in the present study. We compared patients who visited their dentist at least once a year (group A) with those who visited their dentist less than once a year (group B).

**Results:**

Patients in group A had significantly better health-related quality of life after tumor treatment than patients in group B. Patients in group A also had a smaller tumor size and less lymph node metastasis and lost fewer teeth during the treatment. This resulted in better prosthetic rehabilitation and better psychological status after tumor treatment.

**Conclusions:**

Dentists play an important role in the early recognition of oral cancer. This study should encourage dentists to take a more active role in oral cancer prevention.

## 1. Introduction

The incidence of oral squamous cell carcinoma (OSCC) is within the top 10 of all cancer entities [1]. The use of tobacco and excessive alcohol consumption are estimated to account for about 90% of all OSCCs [2, 3]. In addition, a relationship with human papillomavirus (HPV) has been shown in a number of studies demonstrating DNA from HPV, particularly with oropharyngeal carcinoma. The possibility of sexual transmission has thus been raised [4]. There is also evidence that average and poor oral hygiene and inadequate dental status are independent risk factors for OSCC and oropharyngeal squamous cell carcinoma, irrespective of tobacco and alcohol consumption [5–7].

Despite the progress in both research and therapy, the overall 5-year survival rate of OSCC is approximately 50–60% and has not changed significantly in recent years [8]. Furthermore, survival and cure rates vary considerably for the different tumor stages (I–IV) [9]. Patients with stage I tumors have a good prognosis, with cure rates of approximately 80%, while patients with stage II tumors have lower cure rates of approximately 65%. Many patients with advanced tumor stages (III and IV) have 5-year survival rates of approximately 16–50% and cure rates of approximately 30%. With the presence of distant metastasis, survival duration decreases to approximately 4 months [1, 10]. Therefore, early detection of OSCC is the single most important factor that influences prognosis.

Late diagnosis results in not only higher use of healthcare resources but also lower survival rates, function, and quality of life (QOL) among survivors [11–14]. Also, the more specific health-related quality of life (HRQOL) significantly decreases with increasing tumor stage [15]. Dentists, oral hygienists, and other healthcare providers play an important role in oral cancer prevention, by providing oral examinations and detecting early oral cancers. Currently, there is insufficient evidence to recommend a cost-intensive OSCC screening program for the whole population. Therefore, many authors recommend a targeted population approach that includes screening high-risk patients, such as severe smokers or heavy drinkers, for OSCC. This could shift the stage at detection and reduce overall mortality [10, 16–18].

Many dentists are currently reluctant to tell their patients they are performing an oral mucosal examination and often try to avoid the word cancer. Therefore, patients are often unaware of the oral cancer screening and remain uninformed of the particular risks for developing oral cancer [19]. In this large multicenter study, we examined the relationship between regular dental visits and OSCC-related HRQOL. Our aim was to demonstrate that regular dental visits could identify cancer earlier (lower tumor stage) and therefore lead to a higher posttreatment HRQOL. This would emphasize the dentist's role in oral cancer prevention, without the implementation of specialized screening programs, and could help dentists to recognize their valuable role in oral cancer prevention.

## 2. Materials and Methods

### 2.1. Data Source

The sample for analyses consisted of usable, retrospective data for 1,652 patients with OSCC who were treated at 38 hospitals in Germany, Austria, and Switzerland; these data were collected during the DÖSAK Rehab Study. Patients included were those who had been diagnosed with and undergone surgery for OSCC, with or without other adjuvant therapies, at least 6 months before completing the questionnaire. All participants in the DÖSAK Rehab Study provided informed consent, and the current study was approved by the institutional review board of the Ruhr-Universität Bochum. During the DÖSAK Rehab Study, the Bochum Questionnaire on Rehabilitation and a physician questionnaire (*n* = 1,489) were distributed, comprising 147 items in 7 categories: demographics, health behavior, course of disease before surgery, course of disease after surgery, postoperative care, and coping with disease [20].

### 2.2. Patient Questionnaire

The patients were asked about their condition and symptoms at three time points in their treatment process: before treatment (*t*1), immediately after the operation (*t*2), and at least 6 months after the surgical intervention (*t*3). We only used *t*3 for our evaluation.

The patient questionnaire included a list of 19 impairments, which were identified by oral and maxillofacial surgeons as the cause of most symptoms for patients with head and neck cancer, as a result of either the disease itself or subsequent treatment; these included restricted movement of the shoulder/arm, mandible, and neck, as well as difficulties with ingestion and swallowing. The extent/severity of each of these impairments was rated using a five-level Likert scale (none, weak, medium, strong, and very strong).

In addition, more specific impairments were assessed via the patient questionnaire, such as the effects of scarring and swelling; pain in the oral cavity, the temporomandibular joint, and neck and shoulder region; and numb regions in the area of operative treatment or paralysis of the facial muscles.

Data on the psychological status of each patient were collected using short-form standardized tests, including the State-Trait Anxiety Inventory (STAI) for anxiety [21], the five-level depressive scale by von Zerssen (DS) for depressive state [22], and the Freiburg Questionnaire of Coping with Illness (FQCI) for illness processing [23].

The STAI measures relatively stable interindividual differences in the tendency to evaluate situations as threatening and to react with an increasing state-anxiety. The STAI, with its two subscales, was used to document both situation-specific and enduring anxiety. In the present study, the second scale (Trait) was particularly appropriate because of its temporal and situational independency (reliability). The DS measures the presence and, if necessary, the intensity of psychopathologic symptoms in terms of depressive, anxious, or nervous displeasure without giving a clear nosologic diagnosis. With its broad spectrum, the FQCI measures clinically relevant mechanisms of illness coping on cognitive, emotional, and actional levels.

The results for STAI, DS, and FQCI were classified for comparisons. We defined a cut-off point for each measurement (STAI 8/4, DS 8/4, and FKV 10/4); values below this point were classified as low or not pathological, and values above this point were classified as high or pathological.

### 2.3. Physician Questionnaire

The physician questionnaire provided an overview of medically relevant patient data, such as tumor location, tumor size, treatment and reconstructive technique, and type of lymphadenectomy. All surveyed patients were treated surgically.

### 2.4. Measurements and Evaluations

Patients were divided into two groups based on their regular dental visits. Group A visited their dentist ≥1 time per year, and group B visited their dentist <1 time per year.

Tumor size and lymph node status were classified according to the Union for International Cancer Control (UICC) classification for malignant tumors (1987).

QOL was measured on a scale of 0 to 100, where 0 is very bad and 100 is very good, based on the question “Estimate your own actual QOL on a scale of 0 to 100.” The responses for QOL after tumor therapy were divided into three groups: unsatisfied (0–50), rather satisfied (51–80), or very satisfied (81–100). The HRQOL in groups A and B was measured based on QOL and the 7 impairments that had the highest correlations with dental visits.

### 2.5. Statistical Analysis

Statistical analysis was performed using SPSS version 22 (IBM Corp., Armonk, NY, USA), including descriptive statistics, cross-tabulations with Pearson's chi-square tests, and Kendall's tau-b. In addition to the groups based on regular dental visits, we also divided the patients into two groups based on remaining teeth after tumor treatment: those with remaining teeth and those who were edentulous. A *p* value < 0.05 was considered statistically significant.

## 3. Results

### 3.1. Patient Characteristics

Of the 1,652 patients (413 [25%] women, 1,239 [75%] men), 911 patients received surgical treatment only, 502 underwent additional radiotherapy, 78 received additional chemotherapy, 131 underwent additional radio/chemotherapy, and data were not available for 30 patients. Regarding age, 53 patients were ≤40 years old, 829 patients were 41–60 years old, 594 patients were 61–75 years old, and 114 patients were >75 years old.

### 3.2. Dental Visits

**Figure 1:**
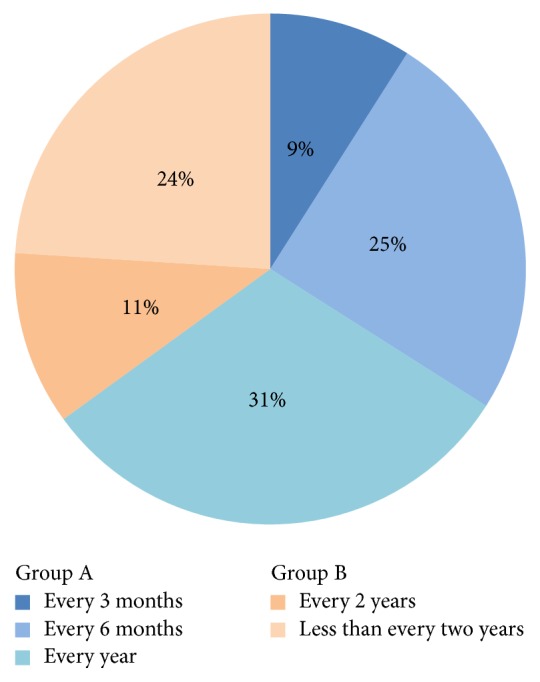
Frequency of dental visits for 1,607 patients with oral squamous cell carcinoma.

Information regarding dental visits was provided by 1,607 patients (Figure 1): 65% (*n* = 1,049) visited ≥1 time per year (group A), and 35% (*n* = 558) visited <1 time per year (group B). Compared with group B, group A consisted of significantly more women than men, more white-collar workers (*n* = 371) than blue-collar workers (*n* = 384), and more nonsmokers or nondrinkers (all, *p* < 0.05). The frequency of dental visits did not differ by age.

### 3.3. Tumor Size and Lymph Node Status

Tumor size was significantly larger, and lymph node metastasis was present significantly more often in group B than in group A (*p* = 0.001 for tumor size and *p* = 0.001 for lymph node metastasis, Table 1).

### 3.4. Tooth Loss and Prosthetic Rehabilitation

Patients in group B lost 10 teeth throughout the tumor treatment significantly more often and were edentulous significantly more often than the patients in group A (both *p* < 0.05, Table 2). Edentulous patients experienced postoperative impairment significantly more often, including numb regions in the lower lip and chin region (*p* < 0.05), paresis at the corner of the mouth (*p* < 0.05), and uncontrollable salivation at the angle of the mouth (*p* < 0.05). Patients in group A could be rehabilitated with a partial prosthesis in the upper and lower jaws significantly more often than patients in group B (*p* < 0.05).

### 3.5. Quality of Life and Health-Related Quality of Life

The HRQOL in groups A and B was measured based on QOL and the 7 impairments that had the highest correlations with dental visits: shoulder-arm-mobility (correlation coefficient [CC] 0.073), speech intelligibility for foreigners (CC 0.069), tongue mobility (CC 0.065), speech intelligibility for relatives (CC 0.058), mouth aperture (CC 0.047), mandible mobility (CC 0.047), and eating/swallowing (CC 0.049). General HRQOL (including impairments in speech, eating/swallowing, opening of mouth, shoulder/arm movement, general condition, breathing, and appearance) was significantly lower in group B than in group A (*p* < 0.05). Patients with remaining teeth described significantly fewer impairments after treatment than edentulous patients (Table 3). In addition, patients who required rehabilitation with a full prosthesis in either jaw reported significantly lower HRQOL than people with a partial prosthesis (*p* < 0.05).

### 3.6. Social and Psychological Status

Significantly more patients in group B experienced fear (*p* < 0.016), depressive tendency (*p* < 0.056), and depressive illness coping (*p* < 0.008). In addition, patients in group B rated their public appearance significantly lower after treatment (*p* < 0.05), and significantly fewer patients in group B were able to return to work in their former job than patients in group A (*p* < 0.05).

## 4. Discussion

The treatment modality, age, and sex distributions in the present study were comparable to those in other studies of OSCC [24–26]. Similar to previous studies, more educated people (white-collar workers) visited their dentist more frequently than less educated people (blue-collar workers) [27, 28]. The return to work by more patients in group A might be related to the association between a higher socioeconomic status and regular dental visits as well as resumption of work after tumor treatment [29, 30].

In contrast to the findings of previous studies, women visited their dentist more often than men [31], and there was no age difference in DV [32, 33]. Nonsmokers and nondrinkers visited their dentist more often, similar to previous studies [34–37]. Tumor size and lymph node status were significantly lower in group A, indicating that patients who regularly visit their dentist are treated at a lower tumor stage, as reported previously [14, 38, 39]. However, it is currently unknown if this results in lower mortality rates or improved survival rates across the population; only one high-quality randomized controlled trial supports this theory [16, 40]. Therefore, many authors agree that it is more important to screen high-risk patients, such as heavy smokers and drinkers, on a regular basis than to perform an opportunistic screening of the whole population [18, 31].

Regarding HRQOL after tumor therapy, HRQOL was significantly higher and psychological status was significantly better in group A, indicating a link between the frequency of dental visits and HRQOL. A link between depressive symptoms and oral health has also been suggested [41]. Patients visiting their dentist on a regular basis lost significantly less teeth during tumor treatment, and fewer of these patients were edentulous after treatment. Therefore, dental rehabilitation with a partial prosthesis was possible. Edentulous patients wearing a full prosthesis had a significantly lower overall QOL than the patients in group A, supporting the findings of Rogers [42]. Even HRQOL was significantly lower in group B; more of these patients complained about numb regions in the lower lip and chin region, paresis of the corner of the mouth, and uncontrollable salivation at the angle of the mouth. The relationship between tooth loss and HRQOL has been reported in a number of studies; even without the presence of a malignant tumor, tooth loss leads to a significant reduction in HRQOL [43, 44].

In our opinion, the better posttreatment dental situation of patients visiting their dentist on a regular basis is related to a lower tumor stage overall at the beginning of tumor therapy as well as better oral health. Therefore, excessive dentoalveolar surgical treatment was not required prior to tumor resection, which normally includes the operative removal of teeth with caries in patients with malignant tumors [45–47].

In conclusion, there are two main benefits of regular dental visits for patients with OSCC: reduced tumor stage and better oral health at the primary presentation. These two benefits result in better posttreatment HRQOL and psychological status in these patients. Therefore, we strongly recommend dental visits at least once a year as opportunistic screening; although mortality rates might not be reduced, HRQOL could be significantly improved for these patients after treatment of OSCC. This should encourage every dentist to perform opportunistic cancer screening and to inform their patients about the obvious benefits of regular dental visits for oral cancer.

## Figures and Tables

**Table 1:** Tumor size and lymph node status in 1,607 patients with oral squamous cell carcinoma, compared by frequency of dental visits.

	Group A	Group B	*p*
*Tumor size*	*n* = 949	*n* = 502	0.001
T1	338 (32)	122 (24)	
T2	368 (35)	216 (43)	
T3	108 (10)	64 (13)	
T4	135 (13)	100 (20)	

*Lymph node status*	*n* = 937	*n* = 491	0.001
N0	607 (65)	267 (55)	
N1	208 (22)	119 (24)	
N2	111 (12)	94 (19)	
N3	11 (1)	11 (2)	

Group A visited their dentist ≥1 time per year, and group B visited their dentist <1 time per year. *p* values for comparison of tumor size (T1/T2 versus T3/4 between groups A and B) and lymph node status (N0 versus N1–3 between groups A and B). The counts may not always add up to the total sample size because of missing values. Values are reported as *n* (%).

**Table 2:** Loss of teeth during treatment for oral squamous cell carcinoma in 1,607 patients, compared by frequency of dental visits.

	Group A	Group B	*p*
*Tooth loss during treatment*	*n* = 974	*n* = 508	
0	321 (33)	189 (37)	
1–5	252 (26)	84 (17)	
6–10	177 (18)	82 (16)	
>10	224 (23)	153 (30)	<0.05

*Presence of teeth after treatment*	*n* = 1,012	*n* = 544	
No teeth	338 (33)	321 (59)	<0.05
Teeth	674 (67)	223 (41)	

Group A visited their dentist ≥1 time per year, and group B visited their dentist <1 time per year. *p* values for comparison of tooth loss (>10) and presence of no teeth after treatment between groups A and B. The counts may not always add up to the total sample size because of missing values. Values are reported as *n* (%).

**Table 3:** Comparisons of the more serious impairments between edentulous patients and patients with remaining teeth after treatment for oral squamous cell carcinoma (*n* = 1652).

Impairment	*p* value	Own teeth *n*	Edentulous *n*	Total *n*	Missing *n*
Intelligibility of speech for strangers	0.001	857	622	1479	173
Intelligibility of speech for family	0.001	866	627	1493	159
Eating/swallowing	0.001	866	628	1494	158
Mobility of tongue	0.001	861	625	1486	166
Mouth aperture	0.001	861	629	1490	162
Mobility of lower jaw	0.001	859	626	1485	167
Mobility of neck	0.001	860	622	1482	170
Shoulder and arm mobility	0.001	863	622	1485	178
Gustatory capability	0.001	861	621	1482	164
Olfactory capability	0.001	858	616	1474	178
Appearance	0.001	861	627	1488	168
Strength	0.001	861	627	1488	179
Appetite	0.001	857	627	1484	169
Breathing	0.001	857	616	1473	192
Pain	0.040	856	627	1483	169
Swelling	0.247	853	607	1460	192
Dryness of mouth	0.001	854	629	1483	169
Halitosis	0.185	845	616	1461	191
Stomach complaints	0.001	856	621	1477	175
